# Supplementary material for: CTCF knockout in zebrafish induces alterations in regulatory landscapes and developmental gene expression
Source: Nat Commun. 2021 Sep 13;12:5415. doi: 10.1038/s41467-021-25604-5 (PMC8438036; doi:10.1038/s41467-021-25604-5)
Supplement: Supplementary file 1 — Supplementary Information [file 41467_2021_25604_MOESM1_ESM.pdf]

CTCF knockout in zebrafish induces alterations in regulatory landscapes and developmental gene expression

Martin Franke[1#], Elisa De la Calle-Mustienes[1#], Ana Neto[1], María Almuedo-Castillo[1], Ibai Irastorza-Azcarate[2], Rafael D. Acemel[1], Juan J. Tena[1], José M. Santos-Pereira[1*] and José L. Gómez-Skarmeta[1†]

[1] Centro Andaluz de Biología del Desarrollo (CABD), Consejo Superior de Investigaciones Científicas/Universidad Pablo de Olavide, 41013 Seville, Spain.

[2] Berlin Institute for Medical Systems Biology, Max-Delbrück Centre for Molecular Medicine, 10115 Berlin, Germany.

[#] These authors contributed equally to this work

[†] Deceased

* Corresponding author:

José M. Santos-Pereira
phone: +34 954 348 687
email: jmsanper1@upo.es

Supplementary Material

13 Supplementary Figures and Legends

1 Supplementary Table

Supplementary Figure 1

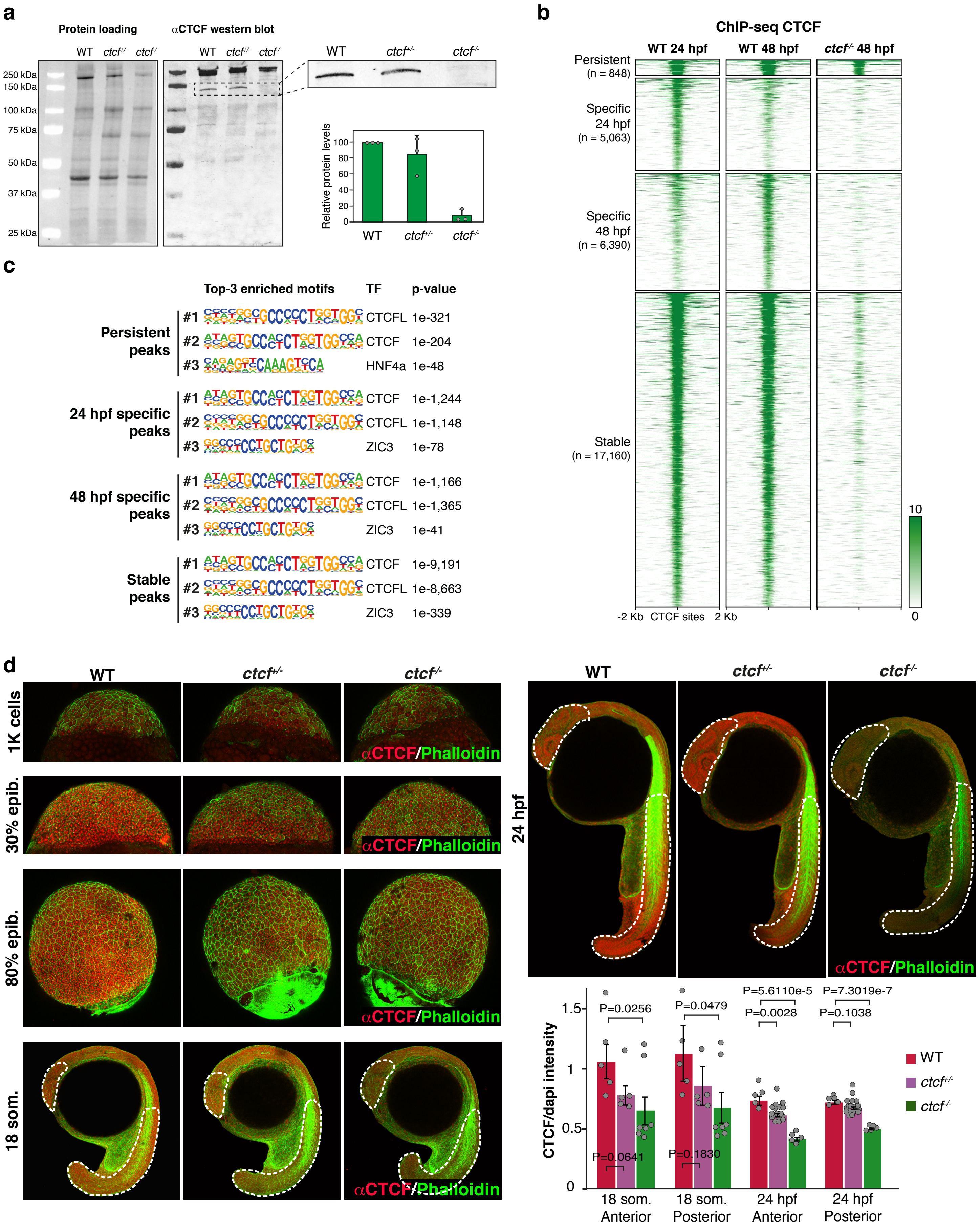

Supplementary Figure 1. CTCF protein and its chromatin binding in *ctcf* knockout zebrafish embryos. a, Western blot of CTCF using an antibody recognizing the N-terminal domain of CTCF (PA5-88115, ThermoFisher Scientific). Left, protein electrophoresis and blot with anti-CTCF of whole-embryo extracts from wild type (WT), *ctcf*$^{+/-}$ and *ctcf*$^{-/-}$ embryos at 48 hours post fertilization (hpf). Right, magnification of the band corresponding to CTCF and protein quantification normalized to protein loading showing average and standard deviation of three independent experiments. Source data are provided as a Source Data file. **b,** Heatmaps of CTCF ChIP-seq in WT embryos at 24 and 48 hpf and in *ctcf*$^{-/-}$ embryos at 48 hpf, showing the signal around the called peaks divided in 4 categories: persistent (peaks called in *ctcf*$^{-/-}$ embryos), specific 24 hpf, specific 48 hpf (peaks called only in WT at either 24 or 48 hpf) and stable (peaks called in WT embryos both at 24 and 48 hpf). **c,** Motif enrichment analyses of the peak categories in **b** showing the top-3 most enriched motifs and their associated P values calculated with a one-sided binomial distribution by HOMER software. **d,** Whole-mount embryo immunofluorescence of CTCF (red) and phalloidin (green) in WT, *ctcf*$^{+/-}$ and *ctcf*$^{-/-}$ zebrafish embryos at the stages of 1,000 cells (1K cells), 30% of epiboly (30% epib.), 80% of epiboly (80% epib.), 18 somites (18 som.) and 24 hpf showing the maternal contribution of CTCF protein. Relative quantification of CTCF/dapi signal from Figure 1b distinguishing between anterior and posterior regions of the embryo (delimited by dashed white lines in embryo pictures) at 18 som. and 24 hpf stages. Average values +/- standard error are shown. Statistical significance was measured using two-sided Student's *t*-test. The number of embryos for WT, *ctcf*$^{+/-}$ and *ctcf*$^{-/-}$ used for quantification are as follows: 18 som. (n=5, n=5, n=7) and 24 hpf (n=5, n=17, n=5). Source data are provided as a Source Data file.

Supplementary Figure 2

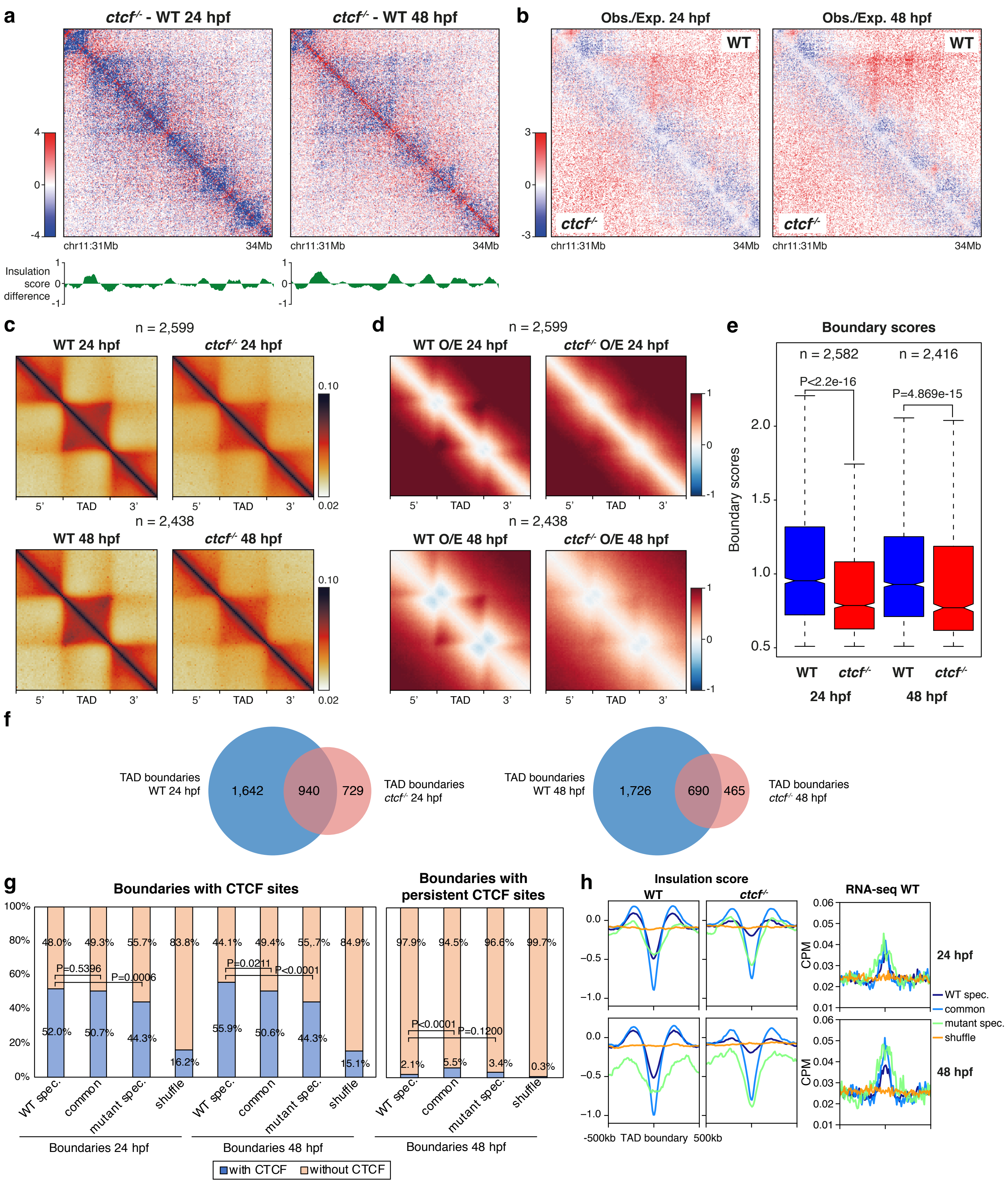

Supplementary Figure 2. Analysis of TAD boundaries in *ctcf* knockout embryos. **a,** HiC normalized contact maps at 10-Kb resolution showing the signal difference between *ctcf*$^{-/-}$ and WT zebrafish embryos at 24 and 48 hpf. A 3-Mb genomic region in chr11 is plotted, aligned with the insulation score difference. **b,** HiC normalized contact maps at 10-Kb resolution showing the observed/expected ratio of WT and *ctcf*$^{-/-}$ zebrafish embryos at 24 and 48 hpf. The same genomic region from **a** is plotted. **c,** Aggregate analysis of normalized HiC signal in WT and *ctcf*$^{-/-}$ embryos at 24 and 48 hpf for the TADs detected in WT embryos at these stages, rescaled and surrounded by windows of the same size. **d,** Aggregate analysis of observed/expected HiC signal in WT and *ctcf*$^{-/-}$ embryos at 24 and 48 hpf for the TADs detected in WT embryos. **e,** Box plots showing the boundary scores of the TAD boundaries called in WT and *ctcf*$^{-/-}$ embryos at 24 and 48 hpf. Box plots represent: center line, median; box limits, upper and lower quartiles; whiskers, 1.5x interquartile range; notches, 95% confidence interval of the median. Statistical significance was assessed using a two-sided Wilcoxon's rank sum test. **f,** Venn diagrams showing the overlap between the TAD boundaries called in WT and *ctcf*$^{-/-}$ embryos at 24 and 48 hpf. For the calculation of these overlaps, TAD boundaries were first extended +/- 1 bin (10 Kb). **g,** Percentage of TAD boundaries containing or not CTCF sites (ChIP-seq peaks) for WT-specific, common and mutant-specific boundaries at 24 and 48 hpf, as well as shuffle controls. Statistical significance was calculated using a two-sided Fisher's exact test. **h,** Average insulation score profiles and normalized RNA-seq signal (counts per million, CPM) around WT-specific, common and mutant-specific boundaries at 24 and 48 hpf, as well as shuffle controls.

Supplementary Figure 3

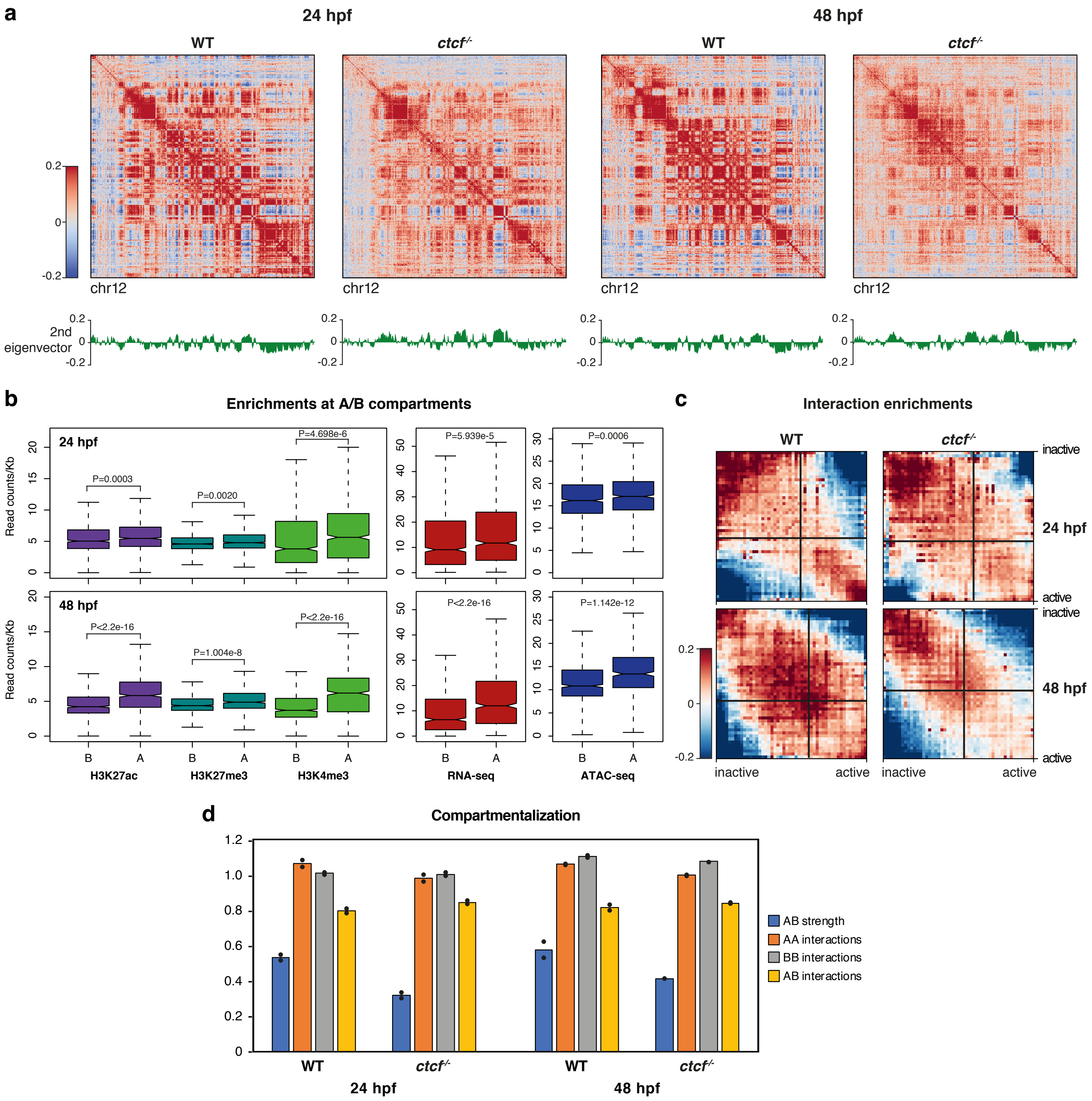

Supplementary Figure 3. Knockout of *ctcf* in zebrafish affects higher-order chromatin architecture. a, Pearson's correlation matrices from HiC data at 100-Kb resolution in WT and *ctcf*$^{-/-}$ embryos at 24 and 48 hpf. The whole chromosome 12 is plotted, together with the 2nd eigenvector demarcating A and B compartments. **b,** Boxplots showing the enrichment of histone marks (H3K27ac, H3K27me3 and H3K4me3), RNA-seq and ATAC-seq used to define the genomic regions demarcated as A and B compartments in WT. Box plots represent: center line, median; box limits, upper and lower quartiles; whiskers, 1.5x interquartile range; notches, 95% confidence interval of the median. Statistical significance was assessed using a two-sided Wilcoxon's rank sum test. **c,** Saddle plots showing the genome-wide interaction enrichments between active and inactive genomic regions from HiC data at 100-Kb resolution in WT and *ctcf*$^{-/-}$ embryos at 24 and 48 hpf. Black lines represent the transition from negative to positive eigenvalues from the 2nd eigenvector in each plot. **d,** Bar plots showing the average interactions between A and B compartments defined by the 2nd eigenvector at 500-Kb resolution of WT and *ctcf*$^{-/-}$ embryos at 24 and 48 hpf. Average and individual data points of two replicates per condition are shown.

Supplementary Figure 4

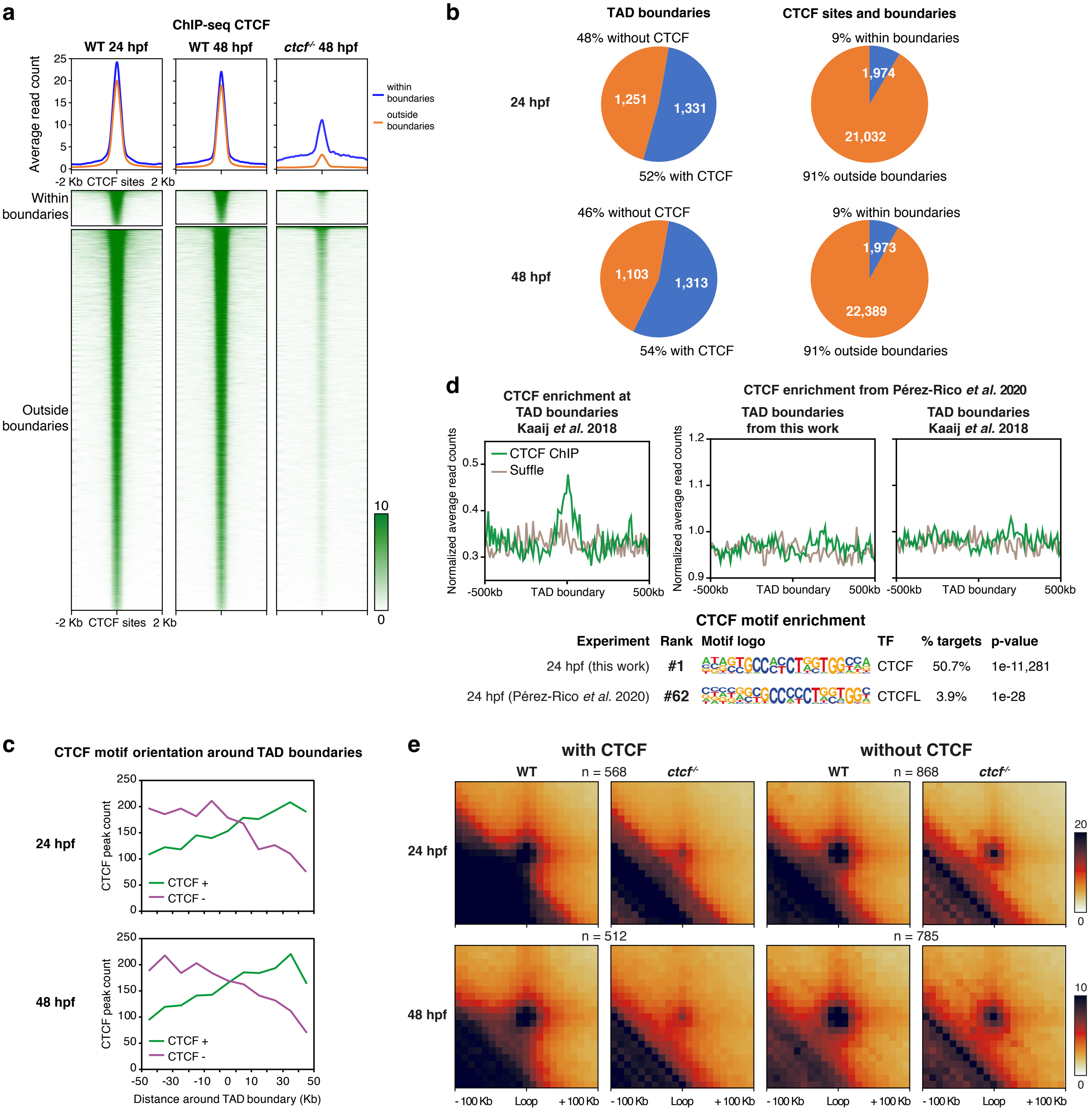

Supplementary Figure 4. CTCF is enriched at TAD boundaries of zebrafish embryos and contributes to the formation of chromatin loops. a, Heatmaps and average profiles of CTCF sites within or outside TAD boundaries. **b,** Pie charts showing the percentage of TAD boundaries overlapping with CTCF sites (left) and the percentage of CTCF sites overlapping with TAD boundaries (right) in WT embryos at 24 and 48 hpf. **c,** CTCF peak count of those peaks containing CTCF motifs located in the positive (CTCF +) or negative (CTCF -) strands around TAD boundaries in WT embryos at 24 and 48 hpf, showing a clear preference for CTCF + motifs in the 3' side of the boundary and for CTCF - motifs in the 5' side of the boundary. **d**, Average CTCF ChIP-seq signal around TAD boundaries (green) and shuffle controls (brown) using the following experiments: left, ChIP-seq from this study and TAD boundaries of zebrafish embryos at 24 hpf from Kaalij *et al.*, 2018; middle, ChIP-seq of HA-tagged CTCF embryos at 24 hpf from Pérez-Rico *et al.*, 2020 and TAD boundaries from this study; right, ChIP-seq from Pérez-Rico *et al.*, 2020 and TAD boundaries from Kaalij *et al.*, 2018. Enrichment of the CTCF motif from ChIP-seq peaks of this study and Pérez-Rico *et al.*, 2020 is shown below. **b, e,** Aggregate peak analysis of chromatin loops called by HiCCUPs with or without CTCF binding in WT and *ctcf*$^{-/-}$ embryos at 24 and 48 hpf.

Supplementary Figure 5

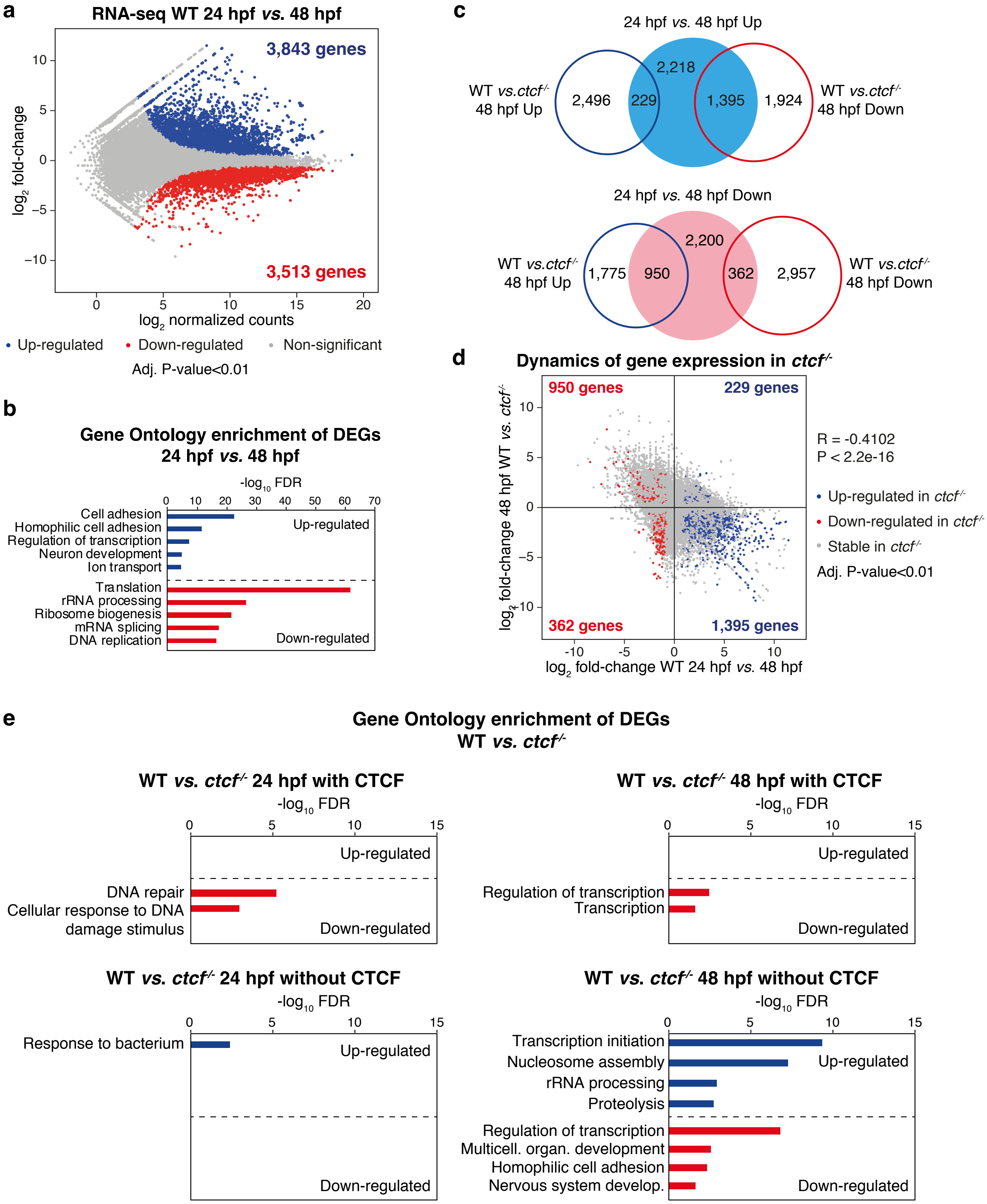

Supplementary Figure 5. CTCF is required for the expression of developmental genes. a, Differential analysis of gene expression in WT embryos between 24 and 48 hpf from RNA-seq data (n = 2 biological replicates per condition). The $\log_2$ normalized read counts of 24-hpf transcripts versus the $\log_2$ fold-change of expression are plotted. Transcripts showing a statistically significant differential expression (adjusted P-value < 0.01) are highlighted in blue (up-regulated) or red (down-regulated). The number of genes that correspond to the up- and down-regulated transcripts are shown inside the boxes. **b,** GO enrichment analyses of biological processes for the up- and down-regulated genes in WT embryos from 24 to 48 hpf. Terms showing an FDR < 0.05 are considered as enriched. **c,** Venn diagrams showing the overlap between the genes up- and down-regulated in WT embryos from 24 to 48 hpf and the genes up- and down-regulated in *ctcf*$^{-/-}$ embryos at 48 hpf (see Fig. 2b). **d,** Scatter plots showing the correlation between the expression fold change of all transcripts in WT embryos from 24 to 48 hpf, and their expression fold change in *ctcf*$^{-/-}$ embryos at 48 hpf. Up- and down-regulated transcripts in *ctcf*$^{-/-}$ embryos are highlighted in blue or red, respectively. **e,** GO enrichment analyses of biological processes for the up- and down-regulated genes in *ctcf*$^{-/-}$ embryos at 24 (left) and 48 hpf (right), distinguishing between those genes with (top) or without (bottom) CTCF binding at their TSS. Terms showing a false discovery rate (FDR) < 0.05 are considered as enriched.

Supplementary Figure 6

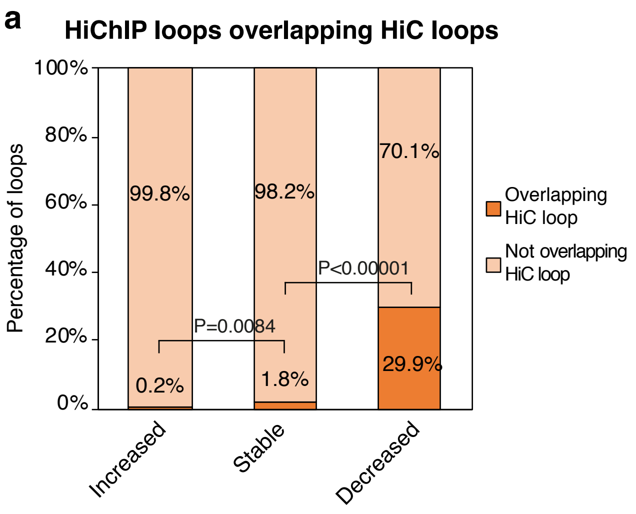

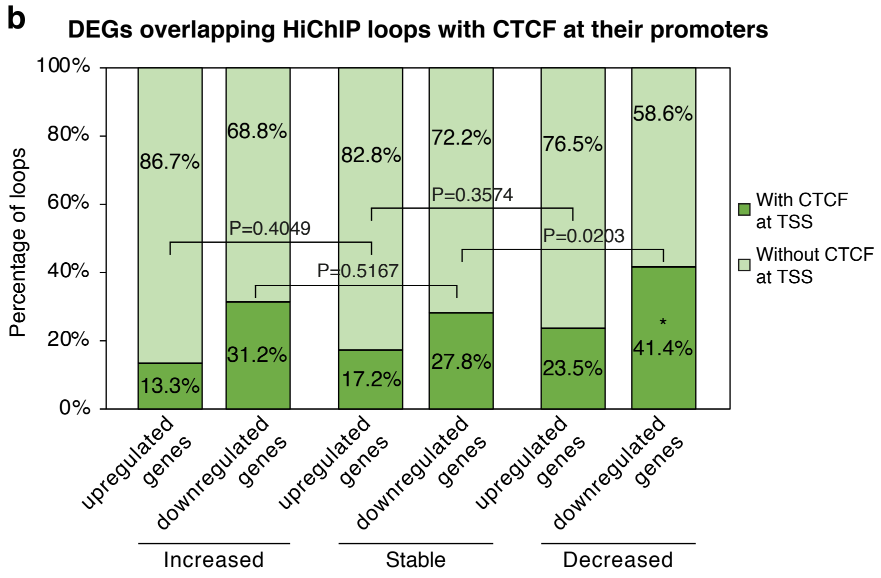

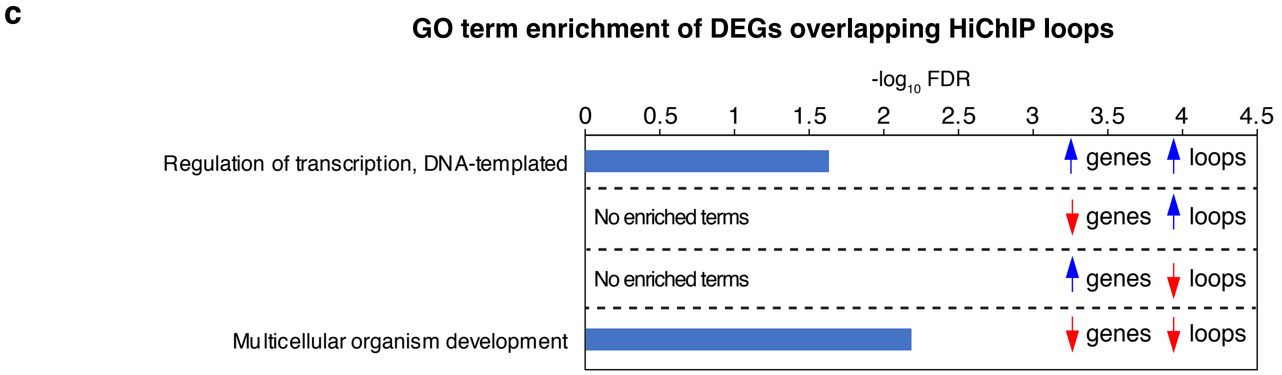

Supplementary Figure 6. Overlap of HiChIP loops with HiC loops and DEGs. a, Percentage of H3K4me3 HiChIP loops overlapping HiC loops called in WT embryos at 48 hpf for increased, stable and decreased HiChIP loops. Statistical significance was assessed using a two-sided Fisher's exact test. **b,** Percentage of H3K4me3 HiChIP loops overlapping the TSS of DEGs bound or not by CTCF. **c,** GO enrichment analyses of biological processes for the DEGs overlapping HiChIP loop anchors for the increased, stable and decreased loops.

Supplementary Figure 7

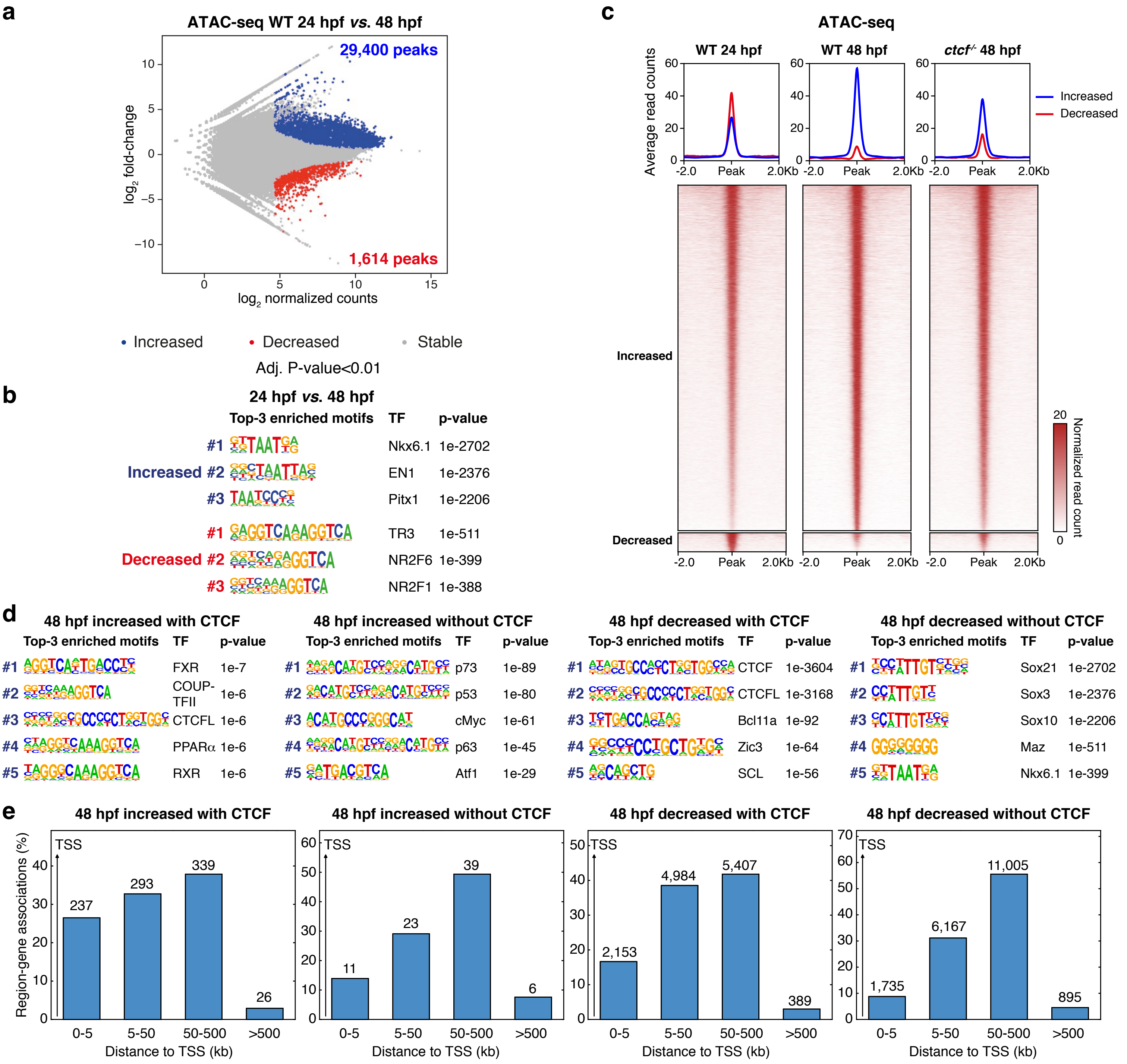

Supplementary Figure 7. CTCF loss affects developmental dynamics of chromatin accessibility. a, Differential analysis of chromatin accessibility in WT embryos between 24 and 48 hpf from ATAC-seq data (n = 2 biological replicates per condition). The $\log_2$ normalized read counts of 24-hpf ATAC peaks versus the $\log_2$ fold-change of accessibility are plotted. Regions showing a statistically significant differential accessibility (adjusted P-value < 0.01, according to the Wald's test performed by the DESeq2 package) are highlighted in blue (increased) or red (decreased). The number of peaks that correspond to the increased and decreased sites are shown inside the boxes. **b,** Motif enrichment analyses for the increased and decreased ATAC peaks in WT embryos from 24 to 48 hpf. The 3 motifs with the lowest p-values are shown for each case. **c,** Heatmaps and average profiles plotting normalized ATAC-seq signal in WT embryos at 24 and 48 hpf and in *ctcf*$^{-/-}$ embryos at 48 hpf for the increased and decreased peaks from (a). **d,** Motif enrichment analyses for the increased and decreased ATAC peaks in *ctcf*$^{-/-}$ embryos at 48 hpf (see Figure 4b) with or without CTCF binding detected by ChIP-seq. The 5 motifs with the lowest p-values (calculated with a one-sided binomial distribution by HOMER software) are shown for each case. **e,** Distribution of distances to the closest TSS using the GREAT tool for the ATAC peaks in d.

Supplementary Figure 8

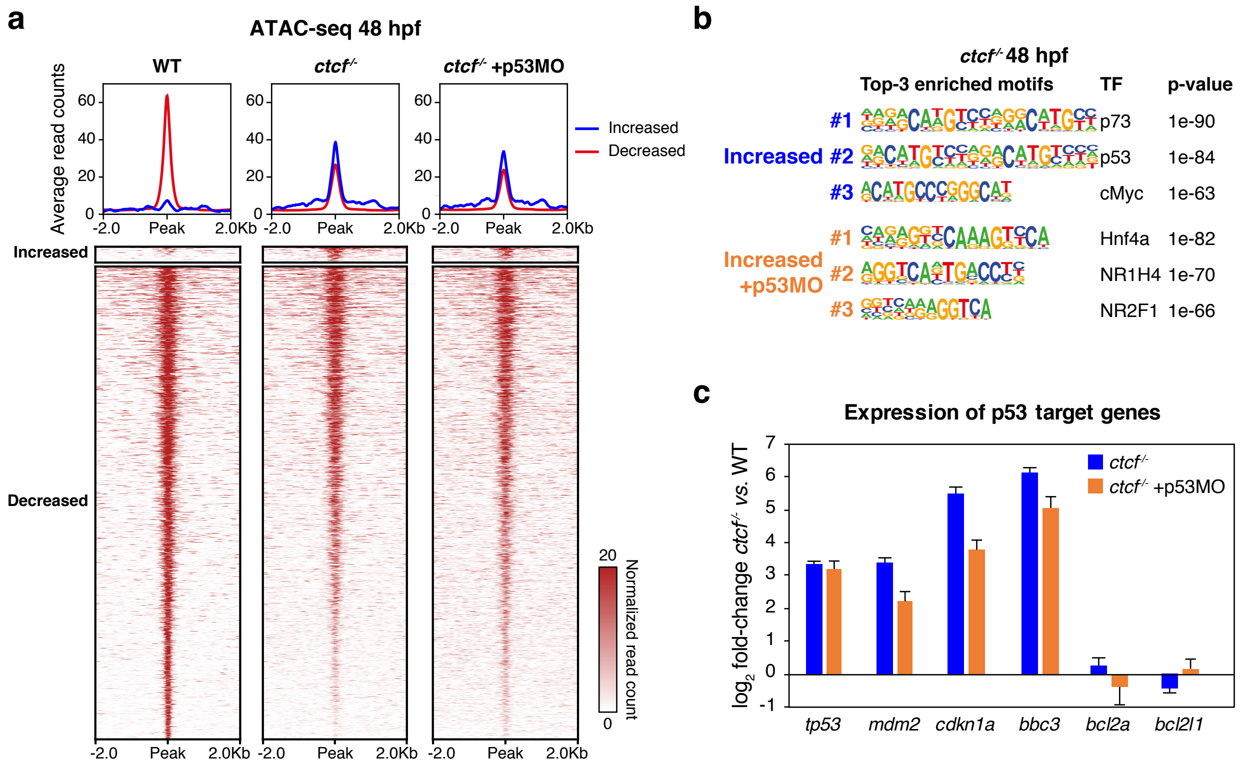

Supplementary Figure 8. The p53 pro-apoptotic response in the absence of CTCF does not suppress defects in chromatin accessibility. a, Heatmaps and average profiles plotting normalized ATAC-seq signal in WT, control *ctcf*$^{-/-}$ and p53 morpholino (p53MO)-injected *ctcf*$^{-/-}$ embryos at 48 hpf for the increased and decreased peaks in control *ctcf*$^{-/-}$ embryos (see Fig. 4b). **b,** Motif enrichment analyses for the increased ATAC peaks in control *ctcf*$^{-/-}$ and p53MO-injected *ctcf*$^{-/-}$ embryos at 48 hpf. The 3 motifs with the lowest p-values (calculated with a one-sided binomial distribution by HOMER software) are shown for each case. **c,** Gene expression fold change from RNA-seq data of the *tp53* gene and the p53 target genes *mdm2*, *cdkn1a*, *bbc3*, *bcl2a* and *bdl2l1*, in control *ctcf*$^{-/-}$ and p53MO-injected *ctcf*$^{-/-}$ embryos at 48 hpf. Average and standard error of the differential expression analysis, which is performed by DESeq2 on the four conditions (WT and *ctcf*$^{-/-}$, injected or not with p53MO) with two biological replicates each, is shown.

Supplementary Figure 9

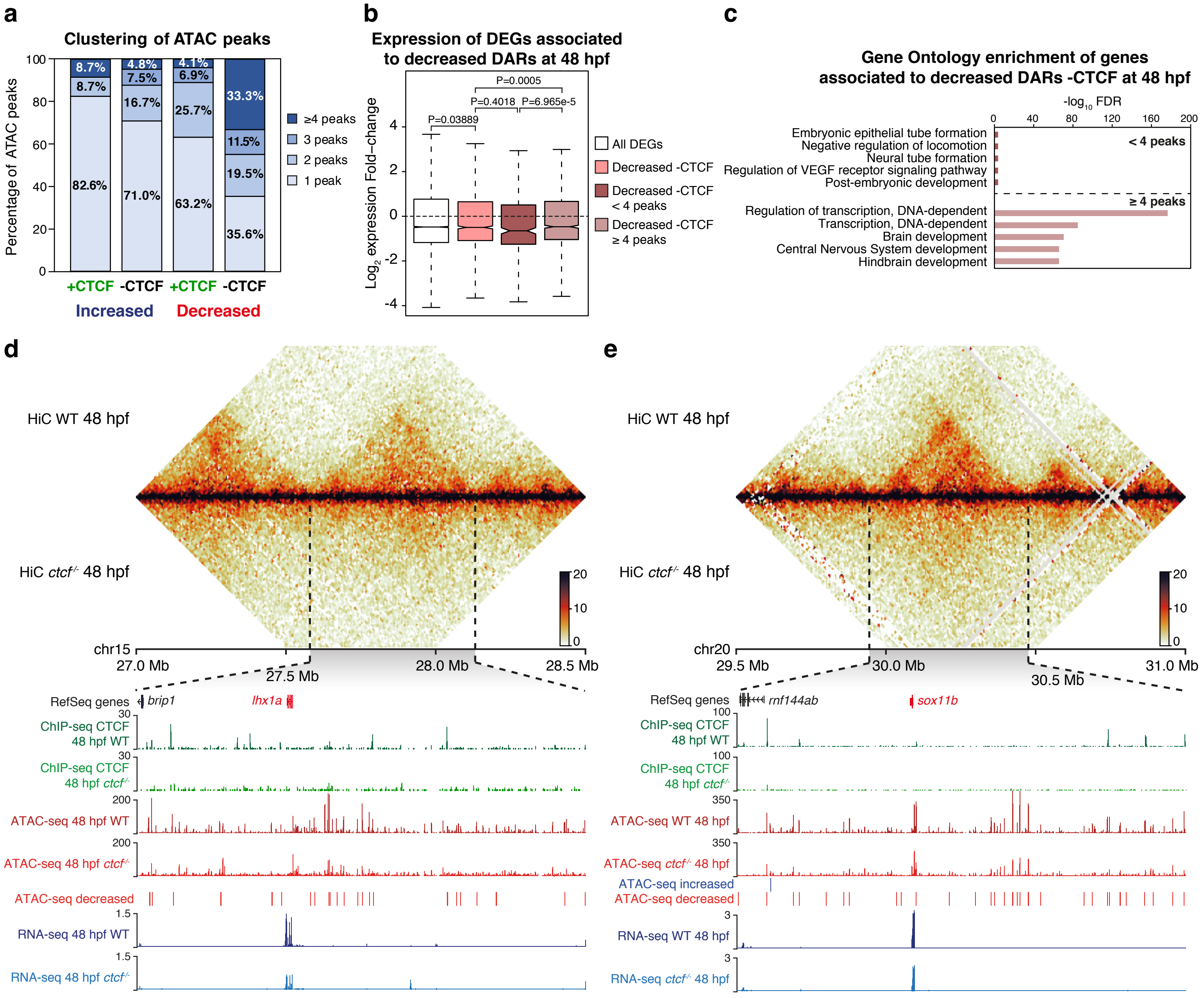

Supplementary Figure 9. CTCF loss reduces accessibility at clustered *cis*-regulatory elements around developmental genes. a, Bar plots showing the level of clustering of the increased and decreased ATAC-seq peaks in *ctcf*$^{-/-}$ embryos at 48 hpf, with or without CTCF binding. Peaks were considered as clustered when located less than 30 Kb from each other. **b,** Box plots showing the expression fold-change in *ctcf*$^{-/-}$ embryos at 48 hpf of all DEGs or only those associated with decreased DARs not overlapping with CTCF sites, grouped in less or more than 4 peaks per cluster. Center line, median; box limits, upper and lower quartiles; whiskers, 1.5x interquartile range; notches, 95% confidence interval of the median. Statistical significance was assessed using a two-sided Wilcoxon's rank sum test. **c,** GO enrichment analyses of biological processes for the genes associated with the decreased DARs in *ctcf*$^{-/-}$ embryos at 48 hpf not overlapping with CTCF sites, grouped in less or more than 4 peaks per cluster. Top-5 terms showing an FDR < 0.05 are plotted. **d-e,** Top, heatmaps showing HiC signal in WT and *ctcf*$^{-/-}$ embryos at 48 hpf in a 1.5-Mb region of chromosomes 15 (d) or 20 (e). Bottom, zoom within the *lhx1a* TAD (d) or the *sox11b* TAD (e) showing tracks with CTCF ChIP-seq, ATAC-seq and RNA-seq at 48 hpf in WT and *ctcf*$^{-/-}$ embryos, as well as ATAC-seq increased and decreased peaks. The down-regulated genes are shown in red.

Supplementary Figure 10

a

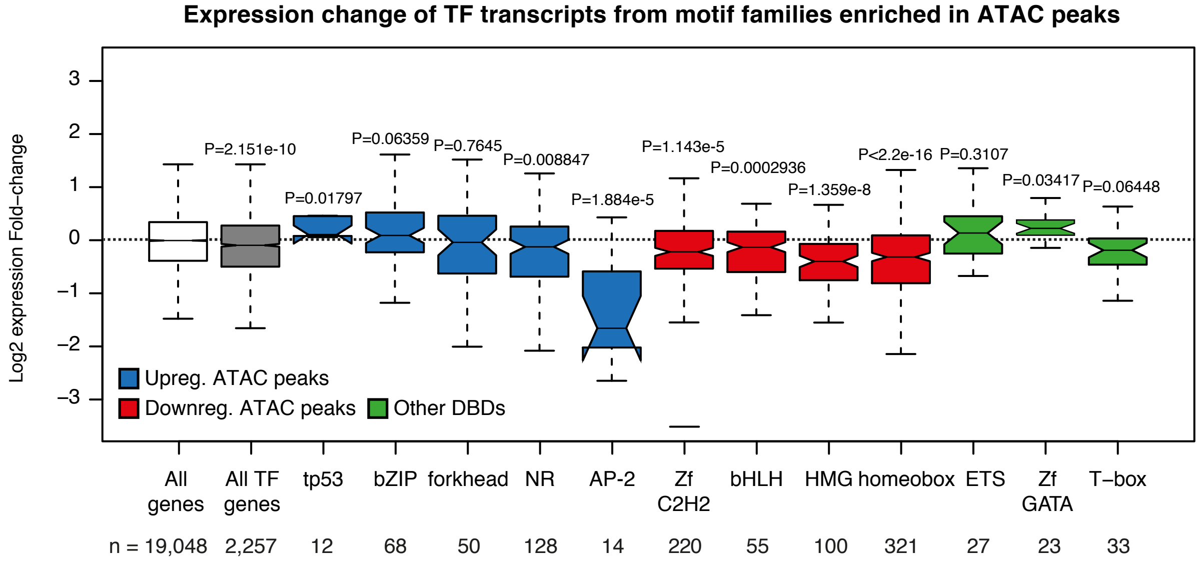

b

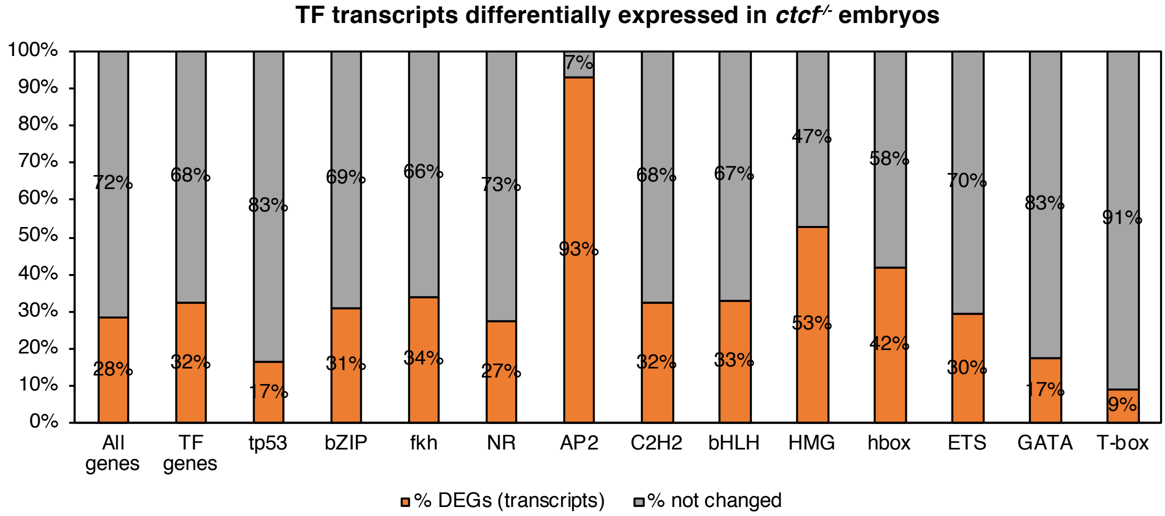

Supplementary Figure 10. Expression of transcription factor gene families in the absence of CTCF. a, Boxplots showing the expression fold change using RNA-seq for the transcription factor (TF) gene families whose motifs are enriched in the DARs at 48 hpf (see Fig. 4b). All transcripts with curated expression in the ZFIN database were plotted and compared with genes at 48 hpf (see Fig. 2b). Center line, median; box limits, upper and lower quartiles; whiskers, 1.5x interquartile range; notches, 95% confidence interval of the median. Statistical significance was assessed using a two-sided Wilcoxon's rank sum test. **b,** Percentage of the transcription factor genes that are differentially expressed in *ctcf*$^{-/-}$ embryos.

Supplementary Figure 11

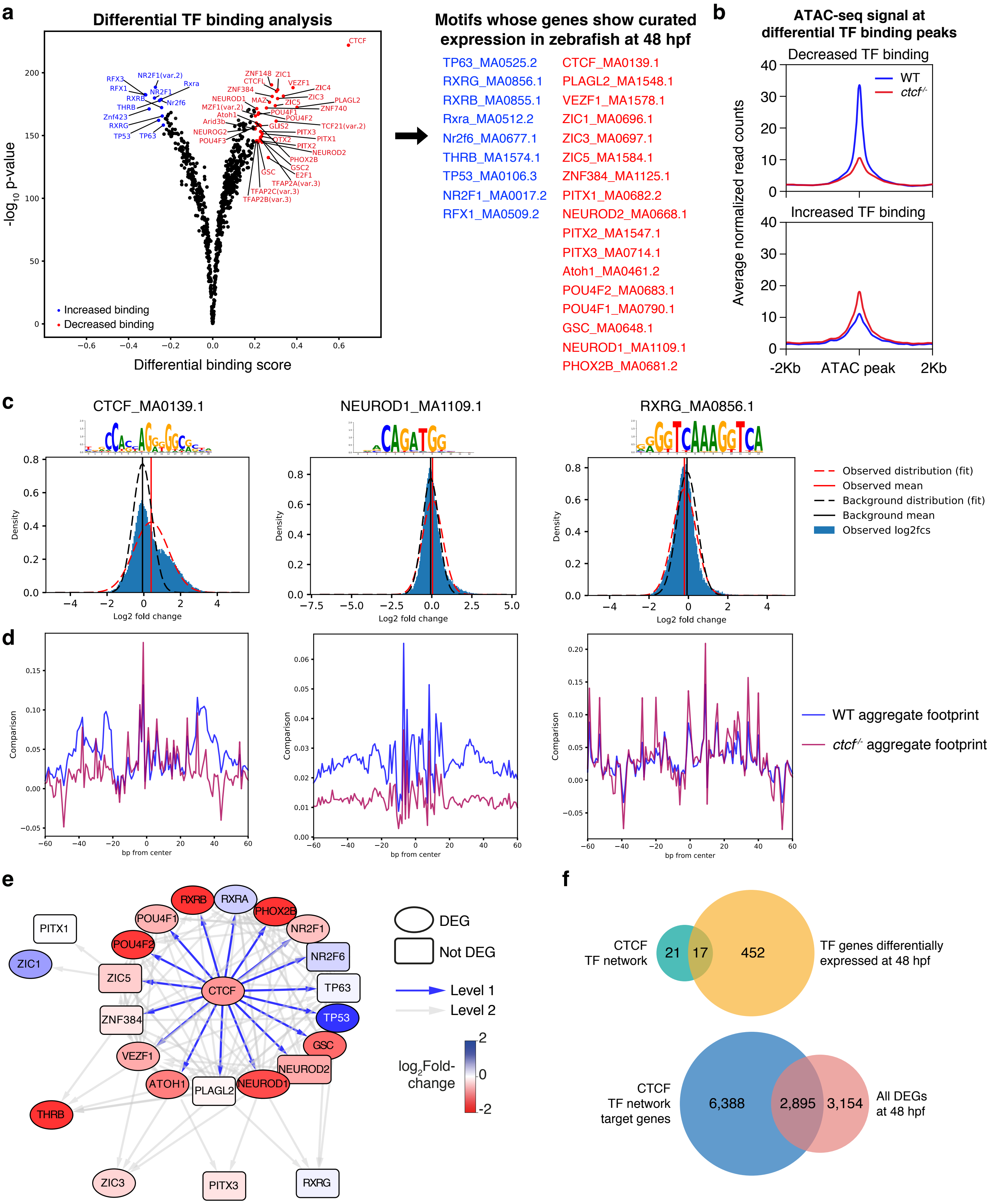

Supplementary Figure 11. Footprinting analyses of ATAC-seq in *ctcf* knockout reveals CTCF transcription factor network. a, Differential transcription factor (TF) binding analysis in WT and *ctcf*$^{-/-}$ embryos using TOBIAS. Left, volcano plot representing the differential binding score versus the -$\log_{10}$ p-value. TF motifs with increased (blue) and decreased (red) binding in mutant embryos are highlighted. Right, TF motifs whose genes show curated expression in zebrafish embryos at 48 hpf according to the ZFIN database. **b,** Average profile showing normalized ATAC-seq signal of WT embryos at 48 hpf for the peaks containing motifs with increased or decreased binding. **c,** Distribution of fold changes for the ATAC peaks containing the motifs of CTCF (decreased), Neurod1 (decreased) and RXRγ (increased) in WT and *ctcf*$^{-/-}$ embryos at 48 hpf. **d,** Aggregate footprint signal for the peaks containing the motifs in c. **e,** TF network of CTCF based on the presence of footprints at gene promoters. Two levels are represented, since the third level did not increase number of involved TFs. Ellipses contain the TF genes that are differentially expressed in *ctcf*$^{-/-}$ embryos, and squares contain those not differentially expressed. Blue and red colors represent the expression fold change according to RNA-seq data at 48 hpf (Fig. 2b). **f,** Venn diagrams plotting the overlap between TF genes of the CTCF network from e and all the TF genes that are differentially expressed in *ctcf*$^{-/-}$ embryos at 48 hpf (top), or the overlap between the putative target genes of the CTCF TF network detected by GREAT and all DEGs at 48 hpf (Fig. 2b).

Supplementary Figure 12

a

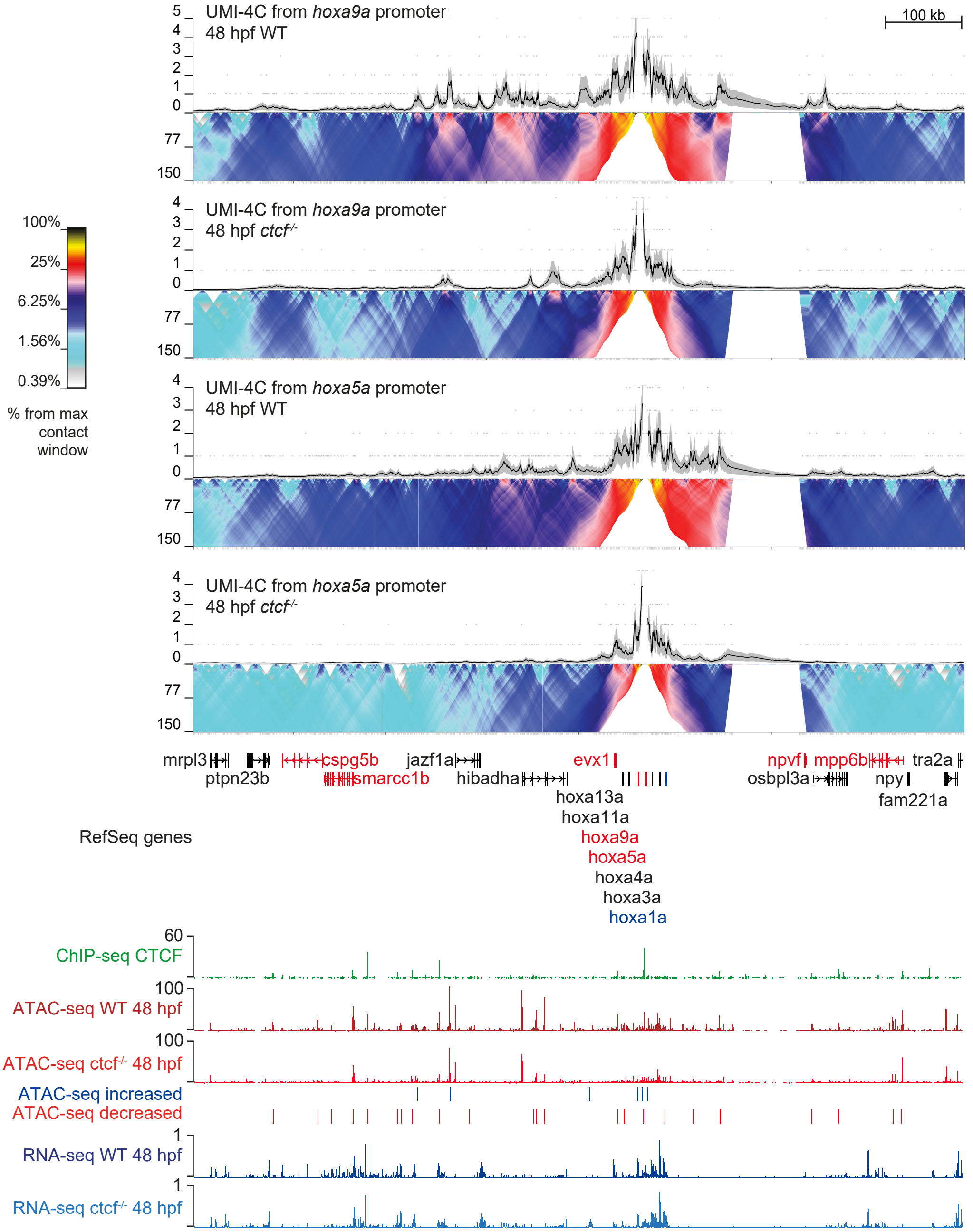

b

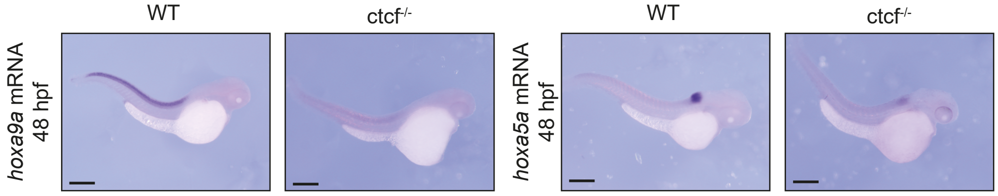

Supplementary Figure 12. CTCF is required for the establishment of regulatory landscapes at the HoxA locus and *hoxa* gene expression. a, Top, UMI-4C assays in WT and *ctcf*$^{-/-}$ embryos at 48 hpf using the *hoxa5a* and *hoxa9a* gene promoters as viewpoints. Black lines and grey shadows represent the average normalized UMI counts and their standard deviation, respectively. Domainograms below UMI counts represent contact frequency between pairs of genomic regions. Bottom, tracks with CTCF ChIP-seq, ATAC-seq and RNA-seq at 48 hpf in WT and *ctcf*$^{-/-}$ embryos, as well as increased and decreased ATAC-seq peaks in *ctcf*$^{-/-}$ embryos. **b,** Whole-mount *in situ* hybridization of the *hoxa5a* and *hoxa9a* genes in WT and *ctcf*$^{-/-}$ embryos at 48 hpf. Anterior is to the right and scale bars represent 500 μm.

Supplementary Figure 13

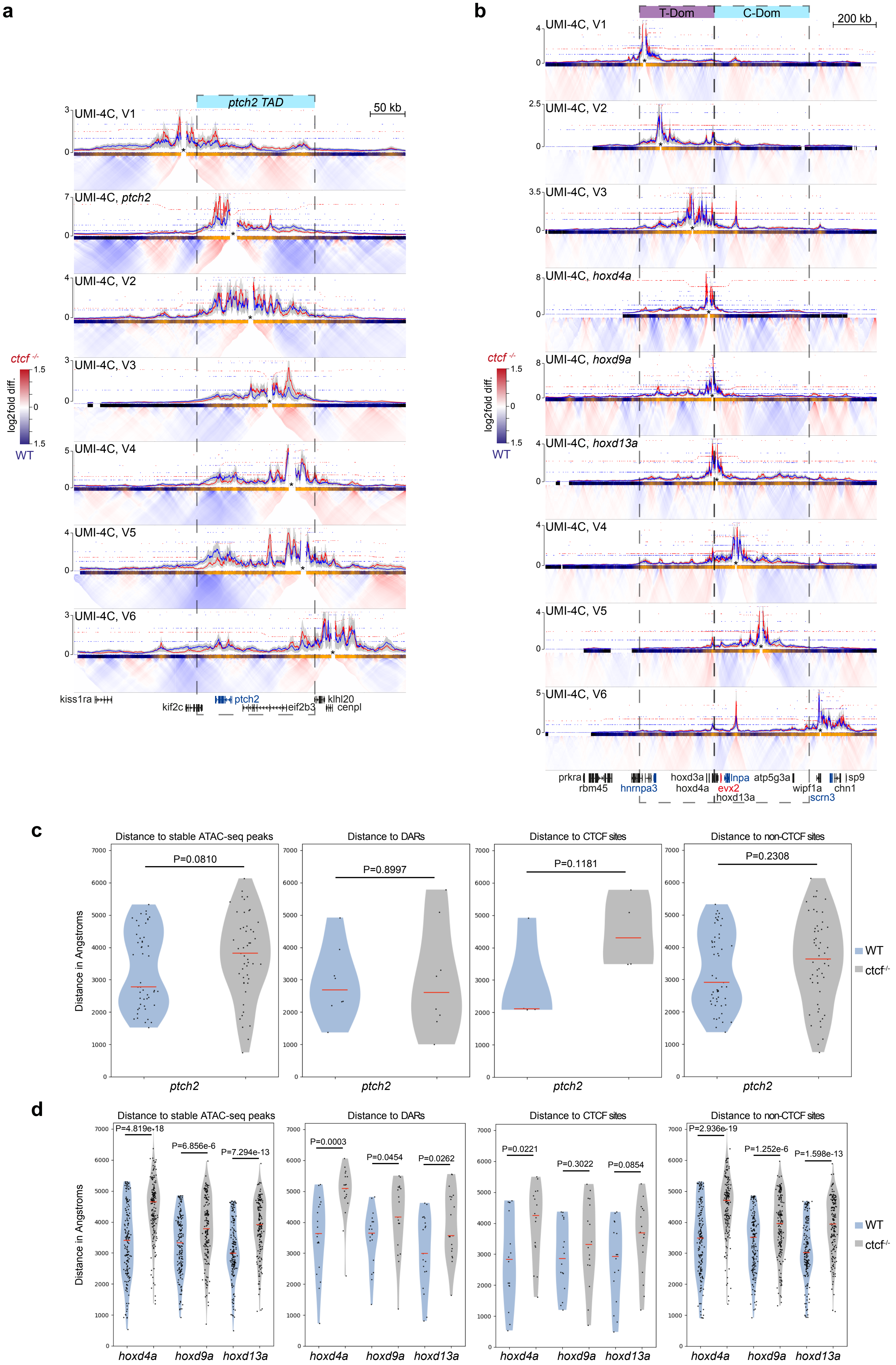

Supplementary Figure 13. Enhancer-promoter distances at the *ptch2* and HoxD loci are decreased upon CTCF loss. a-b, UMI-4C tracks showing the differential contacts between WT and *ctcf*$^{-/-}$ embryos at 48 hpf for all viewpoints used to model the *ptch2* (a) and HoxD (b) loci. T-Dom and C-Dom represent the telomeric and centromeric TADs, respectively, flanking the HoxD locus. **c-d,** Violin plots showing the distances between different subsets of ATAC-seq peaks in the *ptch2* (c) and HoxD (d) loci and *ptch2*, *hoxd4a*, *hoxd9a* and *hoxd13a* promoters. Statistical significance was assessed using a two-sided Student's *t*-test. *, P<0.05; ***, P<0.001; n.s., non-significant.

Supplementary Table 1 – Primer sequences (5'-to-3') used in this study.

Primer name	Primer sequence
sgRNA_CTCFexon4	AATACGACTCACTATAGGAGTTACACTTGCCCACGCGTTTTAGAGCTAGAA
sg_RNACTCFexon5	TAATACGACTCACTATAGGCATGGCCTTTGTCACCAGGTTTTAGAGCTAGAA
sgRNA_univ	AAAAGCACCGACTCGGTGCCACTTTTTCAAGTTGATAACGGACTAGCCTTATTTTAACTTGCTATTTCTAGCTCTAAAAC
genotyping_CTCFpF	CAAGCTGCGCTACAACACAG
genotyping_CTCFpR	CTCCTGTGTGGGAGCGAATG
ISH_probe_ptch2F	TCCTGTGCTGTTTCTACAGG
ISH_probe_ptch2R	GGATCCATTAACCCTCACTAAAGGGAATGCGCAGAACAAGTTATAGG
ISH_probe_hoxa5aF	GGCGTGGACTATCCCTTAC
ISH_probe_hoxa5aR	GGATCCATTAACCCTCACTAAAGGGAAGGAGGCCAATCACACCTTAC
ISH_probe_hoxa9aF	CCCTTCCCTCTACCTTTTCC
ISH_probe_hoxa9aR	GGATCCATTAACCCTCACTAAAGGGAAGAAGGTCAACAGACCATGAGG
ISH_probe_hoxc1aF	GTCTGTGGATGGAGTTTCG
ISH_probe_hoxc1aR	GGATCCATTAACCCTCACTAAAGGGAAGGTGCTTTAACGGTACGTG
umi4C-ptch2-US	CATCAAACCACCCTTTTCAG
umi4C-ptch2-DS	*GGGCTACCTCTCCAAATGTT
umi4C-hoxd4a-US	TTTCCTACCTTCAGAAATTAATGG
umi4C-hoxd4a-DS	*TCGTACATGGTGAACTCCAA
umi4C-hoxd13a-US	GAGCGTGAATACAACACCACTA
umi4C-hoxd13a-DS	*CCACTAAGTTCATTACAAAGGAGA
umi4C-hoxa9a-US	CAGAAGGCAACTACATGAGAATC
umi4C-hoxa9a-DS	*TCCGACAATTTGGTCAGC
umi4C-hoxa5a-US	AAATTGGAAAGCAGCGGAGA
umi4C-hoxa5a-DS	*CAGCGGAGACTAACTTTCAGAG
umi4C-ptch_v1-US	TGCAGAAGAGAGATTTTGAGGT
umi4C-ptch_v1-DS	*GAGATTTTGAGGTAAGCAGCAC
umi4C-ptch_v2-US	AGAAAACAAGCACAGCCAAC
umi4C-ptch_v2-DS	*CGTGCATGAAGAGTGAAAGA
umi4C-ptch_v3-US	TCGACCATTTCCTCTTTCAC
umi4C-ptch_v3-DS	*TCCCGGAGGTCTGTATTATTT
umi4C-ptch_v4-US	CAAACACAAACCACACTGAGC
umi4C-ptch_v4-DS	*CTGGCCCTGCGAATGTTTA
umi4C-ptch_v5-US	TGGTCTGCACATGTAACACC
umi4C-ptch_v5-DS	*GTCTGTGAGTGTAGGTGTGTG
umi4C-ptch_v6-US	GAAAGCGGGATGTTCAAAT
umi4C-ptch_v6-DS	*TTTTTCGCTCGGTGTGTT

umi4C-hoxd_v1-US	ACATCATTTGCCCTTTGAAC
umi4C-hoxd_v1-DS	*TCATGTCAAATGTGTGTCACC
umi4C-hoxd_v2-US	CCTCTACTGACCACAACTGGA
umi4C-hoxd_v2-DS	*GCACATAAACAAAACGGAAAA
umi4C-hoxd_v3-US	CCGGTTTGAGTGTAAGTGTGT
umi4C-hoxd_v3-DS	*GCTGCAGTGGAGGTGAAG
umi4C- hoxd9a-US	AAGCCCGTTAAAGAGATATGGT
umi4C- hoxd9a-DS	*GGTTTGCGACTGGCTCTAT
umi4C-hoxd_v4-US	CTCAGTGCTTCCCCTCAAC
umi4C-hoxd_v4-DS	*TCCTGGAAGTCATCAGAGCA
umi4C-hoxd_v5-US	ATTTGTCCTGGAAGCATTGA
umi4C-hoxd_v5-DS	*TTGCGGCGGATGTTTTCA
umi4C-hoxd_v6-US	GGGAAGCTAGGCACTCTAGG
umi4C-hoxd_v6-DS	*GGAGGGTCTGGGACTAAAGA
(*) P5 adapter 5' of umi-DS primers	AATGATACGGCGACCACCGAGATCTACACTCTTTCCCTACACGACGCTCTTCCGATCT
