# Supplementary material for: CTCF knockout in zebrafish induces alterations in regulatory landscapes and developmental gene expression
Source: Nat Commun. 2021 Sep 13;12:5415. doi: 10.1038/s41467-021-25604-5 (PMC8438036; doi:10.1038/s41467-021-25604-5)
Supplement: Supplementary file 3 — Reporting Summary [file 41467_2021_25604_MOESM3_ESM.pdf]

nature research

Corresponding author(s): José M. Santos-Pereira

Last updated by author(s): Jul 9, 2021

Reporting Summary

Nature Research wishes to improve the reproducibility of the work that we publish. This form provides structure for consistency and transparency in reporting. For further information on Nature Research policies, see our Editorial Policies and the Editorial Policy Checklist.

Statistics

For all statistical analyses, confirm that the following items are present in the figure legend, table legend, main text, or Methods section.

n/a	Confirmed	
☐	☒	The exact sample size (*n*) for each experimental group/condition, given as a discrete number and unit of measurement
☐	☒	A statement on whether measurements were taken from distinct samples or whether the same sample was measured repeatedly
☐	☒	The statistical test(s) used AND whether they are one- or two-sided<br>*Only common tests should be described solely by name; describe more complex techniques in the Methods section.*
☒	☐	A description of all covariates tested
☐	☒	A description of any assumptions or corrections, such as tests of normality and adjustment for multiple comparisons
☐	☒	A full description of the statistical parameters including central tendency (e.g. means) or other basic estimates (e.g. regression coefficient) AND variation (e.g. standard deviation) or associated estimates of uncertainty (e.g. confidence intervals)
☐	☒	For null hypothesis testing, the test statistic (e.g. F, t, r) with confidence intervals, effect sizes, degrees of freedom and P value noted<br>*Give P values as exact values whenever suitable.*
☒	☐	For Bayesian analysis, information on the choice of priors and Markov chain Monte Carlo settings
☒	☐	For hierarchical and complex designs, identification of the appropriate level for tests and full reporting of outcomes
☒	☐	Estimates of effect sizes (e.g. Cohen's d, Pearson's r), indicating how they were calculated

Our web collection on statistics for biologists contains articles on many of the points above.

Software and code

Policy information about availability of computer code

Data collection	We have used the software ImageLab v6.0.1 (Bio-Rad) for western blots image acquisition and protein quantification, and ZEN Black software 2.1 SP3 version for immunofluorescence imaging acquisition.
Data analysis	For data analyses, we used the following packages and tools (all the relevant references and versions are also detailed in the Methods section): ImageJ v2.1.0/1.53c, CRISPRscan, STAR v2.5.3a, HTSeq v0.8.0, DESeq2 v1.18.1, R v3.4.3, DAVID v6.8, Bowtie2 v2.3.5, Samtools v1.9, Bedtools v2.29.2, MACS2 v2.1.1.20160309, Deeptools v3.5, Homer v4.11, GREAT v3.0.0, TOBIAS v0.12.9, Cytoscape v3.8.2, Juicer Tools v1.13.02, FAN-C v0.9.14, FitHiChIP v9.0., umi4cPackage v0.0.0.9000, 4Cin.<br>Custom code used in this study can be accessed in the following links:<br>https://gitlab.com/rdacemel/hic_ctcf-null<br>https://gitlab.com/rdacemel/pancreasregulome

For manuscripts utilizing custom algorithms or software that are central to the research but not yet described in published literature, software must be made available to editors and reviewers. We strongly encourage code deposition in a community repository (e.g. GitHub). See the Nature Research guidelines for submitting code & software for further information.

Data

Policy information about availability of data

All manuscripts must include a data availability statement. This statement should provide the following information, where applicable:

- Accession codes, unique identifiers, or web links for publicly available datasets
- A list of figures that have associated raw data
- A description of any restrictions on data availability

The HiC, ChIPmentation, RNA-seq, ATAC-seq and UMI-4C data generated in this study have been deposited in the Gene Expression Omnibus (GEO) database under

accession code GSE156099 [https://www.ncbi.nlm.nih.gov/geo/query/acc.cgi?acc=GSE156099]. The public datasets used in this study are available in the GEO database under accession codes: GSE105013 [https://www.ncbi.nlm.nih.gov/geo/query/acc.cgi?acc=GSE105013] and GSE133437 [https://www.ncbi.nlm.nih.gov/geo/query/acc.cgi?acc=GSE133437]. Source data are provided with this paper. Data for TF binding motifs were obtained from JASPAR database (http://jaspar.genereg.net/).

Field-specific reporting

Please select the one below that is the best fit for your research. If you are not sure, read the appropriate sections before making your selection.

☒ Life sciences ☐ Behavioural & social sciences ☐ Ecological, evolutionary & environmental sciences

For a reference copy of the document with all sections, see nature.com/documents/nr-reporting-summary-flat.pdf

Life sciences study design

All studies must disclose on these points even when the disclosure is negative.

Sample size	The number of embryos used for sample preparation was determined following previously reported literature (Bogdanovic et al. 2012 PMID 22593555; Santos-Pereira et al., 2019 PMID 31296872; Kaaij et al., Cell Rep 2018 PMID 29972771; Mumbach et al. 2016 PMID 27643841), and allowed the extraction of enough biological material to perform the experiment. The number of replicates for each experiment was determined following the standards previously published for the ENCODE project. Sample sizes and number of replicates are specified in the methods section.
Data exclusions	Zebrafish embryos analyzed for CTCF immunofluorescence in Fig. 1b and Suppl. Fig. 1d whose genotyping failed were excluded from the analysis (see Source Data file).
Replication	All experimental findings were reliably reproduced. We used two biological replicates for the experiments performed in this study, unless indicated.
Randomization	Randomization of the samples is not applicable to our study, since no treatment conditions were compared. All comparisons were performed between different embryonic stages or between different genotypes, which do not require randomization.
Blinding	Blinding was not relevant for our study, since all comparisons were performed automatically using statistical software that is not influenced by the investigator.

Reporting for specific materials, systems and methods

We require information from authors about some types of materials, experimental systems and methods used in many studies. Here, indicate whether each material, system or method listed is relevant to your study. If you are not sure if a list item applies to your research, read the appropriate section before selecting a response.

Materials & experimental systems

n/a	Involved in the study	
☐	☒	Antibodies
☒	☐	Eukaryotic cell lines
☒	☐	Palaeontology and archaeology
☐	☒	Animals and other organisms
☒	☐	Human research participants
☒	☐	Clinical data
☒	☐	Dual use research of concern

Methods

n/a	Involved in the study	
☐	☒	ChIP-seq
☒	☐	Flow cytometry
☒	☐	MRI-based neuroimaging

Antibodies

Antibodies used	anti-CTCF zebrafish: generated and kindly provided by Felix Rencillas-Targa (Carmona-Aldana et al. 2018 PMID 29723654) (not commercially available)<br>anti-CTCF N-terminal: PA5-88115, ThermoFisher Scientific<br>anti-H3K4me3: abcam ab8580<br>Alexa FluorTM 555 Goat anti-rabbit: Invitrogen #A32727<br>Alexa Fluor TM 488 phalloidin: Invitrogen #A12379<br>Goat Anti-Rabbit IgG antibody: StarBright Blue 520, Bio-Rad, Cat. # 12005870<br>anti-digoxigenin antibody: 11093274910 Roche<br>Antibody dilutions or amounts used in each experiment are specified in the Methods section.
Validation	anti-CTCF zebrafish: it was validated by previous studies (Carmona-Aldana et al., 2018 PMID 29723654). We also performed immunostaining assays as a validation.

PA5-88115: validated in human, mouse, non-human primates and rats, for WB, IHC, IP, ChIP and ICC. Here, we used it for WB of zebrafish samples, valdiated by the absence of the target protein in homozygous knockout mutants.
ab8580: validated in human and cow, but predicted in a number of species, for PepArr, ChIP, WB, IHC-P and ICC/IF. It was previously validated for ChIP in zebrafish (Bogdanovic et al. 2012 PMID 22593555).
A32727: validated for WB and ICC/IF
A12379: validated for imaging applications.
12005870: validated in human, mouse, rat, goat and bovine, for fluorescent western blot.
11093274910: validated to detect digoxigenin and digoxin with no cross-reactivity with other steroids. Multiple applications, including in situ hybridization assays used in this work (https://www.sigmaaldrich.com/ES/es/product/roche/11093274910).

Animals and other organisms

Policy information about studies involving animals; ARRIVE guidelines recommended for reporting animal research

Laboratory animals	This study only used adult animals to collect zebrafish embryos at different developmental stages derived from AB and Tübingen strains. Reproductive (4-12 months old) male and female animals were used to obtain fertilized eggs. Genotypes used for crossing were either wild type or heterozygous ctcf+/- generated by CRISPR-Cas9.
Wild animals	The study did not involve wild animals.
Field-collected samples	The study did not involve field-collected samples.
Ethics oversight	All experiments involving animals conform national and European Community standards for the use of animals in experimentation and were approved by the Ethical Committees from the University Pablo de Olavide, CSIC and the Andalusian government.

Note that full information on the approval of the study protocol must also be provided in the manuscript.

ChIP-seq

Data deposition

- [x] Confirm that both raw and final processed data have been deposited in a public database such as GEO.
- [x] Confirm that you have deposited or provided access to graph files (e.g. BED files) for the called peaks.

Data access links<br>*May remain private before publication.*	To review GEO accession GSE156099 [https://www.ncbi.nlm.nih.gov/geo/query/acc.cgi?acc=GSE156099]
Files in database submission	For each sample, we submitted the fastq file with raw reads, the normalized bigWig files and the bed file with called peaks.
Genome browser session<br>(e.g. UCSC)	http://genome.ucsc.edu/s/jmsantos/CTCF_zebrafish_for_reviewers

Methodology

Replicates	Two biological replicates of CTCF ChIP-seq at 24 hpf or 48 hpf have been performed.
Sequencing depth	ChIP_ctcf_24h_wt_rep1: 38541128 total reads; 8537554 uniquely mapped reads; 50-bp paired-end reads<br>ChIP_ctcf_24h_wt_rep2: 38045788 total reads; 4345540 uniquely mapped reads; 50-bp paired-end reads<br>ChIP_ctcf_48h_wt_rep1: 206977280 total reads; 1961156 uniquely mapped reads; 50-bp paired-end reads<br>ChIP_ctcf_48h_wt_rep2: 100858304 total reads; 144957 uniquely mapped reads; 50-bp paired-end reads<br>ChIP_ctcf_48h_ctcf_rep1: 102787408 total reads; 4260196 uniquely mapped reads; 50-bp paired-end reads<br>ChIP_ctcf_48h_ctcf_rep2: 87210220 total reads; 1744702 uniquely mapped reads; 50-bp paired-end reads
Antibodies	The zebrafish-specific CTCF antibodies used in this study is described in the Methods section and published in Carmona-Aldana et al. 2018 PMID 29723654
Peak calling parameters	macs2 callpeak -t experiment.bed -f BED -g 1.5e9 -n experiment-name -q 1e-3
Data quality	Peaks were called using a stringent cutoff (FDR<0.001; fold-change>5), and only peaks called in both biological replicates from the same condition were considered
Software	All the software used to collect and analyze the ChIP-seq data is described in the Methods section: Bowtie2 v2.3.5, Samtools v1.9, Bedtools v2.29.2, MACS2 v2.1.1.20160309, Deeptools v3.5
